# Management of keratoconus: an updated review

**DOI:** 10.3389/fmed.2023.1212314

**Published:** 2023-06-20

**Authors:** Rashmi Deshmukh, Zun Zheng Ong, Radhika Rampat, Jorge L. Alió del Barrio, Ankur Barua, Marcus Ang, Jodhbir S. Mehta, Dalia G. Said, Harminder S. Dua, Renato Ambrósio, Darren Shu Jeng Ting

**Affiliations:** ^1^Department of Cornea and Refractive Surgery, LV Prasad Eye Institute, Hyderabad, India; ^2^Department of Ophthalmology, Queen’s Medical Centre, Nottingham, United Kingdom; ^3^Department of Ophthalmology, Royal Free London NHS Foundation Trust, London, United Kingdom; ^4^Cornea, Cataract and Refractive Surgery Unit, Vissum (Miranza Group), Alicante, Spain; ^5^Division of Ophthalmology, School of Medicine, Universidad Miguel Hernández, Alicante, Spain; ^6^Birmingham and Midland Eye Centre, Birmingham, United Kingdom; ^7^Singapore National Eye Centre, Singapore Eye Research Institute, Singapore, Singapore; ^8^Academic Ophthalmology, School of Medicine, University of Nottingham, Nottingham, United Kingdom; ^9^Department of Cornea and Refractive Surgery, Instituto de Olhos Renato Ambrósio, Rio de Janeiro, Brazil; ^10^Department of Ophthalmology, Federal University of the State of Rio de Janeiro (UNIRIO), Rio de Janeiro, Brazil; ^11^Federal University of São Paulo (UNIFESP), São Paulo, Brazil; ^12^Academic Unit of Ophthalmology, Institute of Inflammation and Ageing, College of Medical and Dental Sciences, University of Birmingham, Birmingham, United Kingdom

**Keywords:** artificial intelligence, refractive surgery, contact lens, cornea, corneal cross-linking, corneal transplant, intracorneal ring segment, keratoconus

## Abstract

Keratoconus is the most common corneal ectatic disorder. It is characterized by progressive corneal thinning with resultant irregular astigmatism and myopia. Its prevalence has been estimated at 1:375 to 1:2,000 people globally, with a considerably higher rate in the younger populations. Over the past two decades, there was a paradigm shift in the management of keratoconus. The treatment has expanded significantly from conservative management (e.g., spectacles and contact lenses wear) and penetrating keratoplasty to many other therapeutic and refractive modalities, including corneal cross-linking (with various protocols/techniques), combined CXL-keratorefractive surgeries, intracorneal ring segments, anterior lamellar keratoplasty, and more recently, Bowman’s layer transplantation, stromal keratophakia, and stromal regeneration. Several recent large genome-wide association studies (GWAS) have identified important genetic mutations relevant to keratoconus, facilitating the development of potential gene therapy targeting keratoconus and halting the disease progression. In addition, attempts have been made to leverage the power of artificial intelligence-assisted algorithms in enabling earlier detection and progression prediction in keratoconus. In this review, we provide a comprehensive overview of the current and emerging treatment of keratoconus and propose a treatment algorithm for systematically guiding the management of this common clinical entity.

## 1. Introduction

Keratoconus was first reported by Benedict Duddell in 1736 (1). Following its first description, various terminologies such as prolapses corneae, cornea conica, sugar-loaf cornea, and procidentia corneae, were introduced in the early literature (2). Around a century later, John Nottingham provided the first detailed description of the disease in his landmark publication in 1854 (2, 3). Pickford described the conical cornea as a disease that is “intractable in nature and fatal to vision” and one in which “the pathology and treatment are so little understood.” Around 170 years later, keratoconus remains an enigmatic disease.

Over the past few decades, rapid advancement in diagnosing and managing keratoconus has been observed. Originally described as a rare disease by the National Institute of Health with an incidence of less than 1 per 2,000 people (4), it is now known that keratoconus is much more common than originally thought. The reported prevalence is highly variable from 0.2 per 100,000 in Russia (5) to 33 per 1,000 in Iran (6). A meta-analysis from 15 countries reported a global prevalence of 1.4 per 1,000 (7). A higher prevalence is noted in Asian and Middle Eastern populations. Pediatric populations have a higher prevalence rate, with a reported prevalence rate ranging from 5.2 per 1,000 people in New Zealand to 47.9 per 1,000 people in Saudi Arabia (8, 9). In addition, it is one of the most common indications for keratoplasty in many countries (10, 11). Nonetheless, some countries have reported a decreasing trend in the number of keratoplasty for keratoconus in view of the implementation of corneal cross-linking (12, 13).

Etiology of keratoconus is multifactorial, with environmental and genetic factors playing important roles (14, 15). Atopy, eye rubbing, and exposure to ultraviolet rays are some of the recognized risk factors. Familial aggregation of the disease has been noted in several studies indicating a genetic transmission (16). In the Collaborative Longitudinal Evaluation of Keratoconus (CLEK) study, around 13.5% of the patients reported a positive family history (17). The most common mode of inheritance described is autosomal dominant with incomplete penetrance and variable expression (18). However, a study based on a segregation analysis on 95 families suggested a possibility of autosomal recessive inheritance (19). Offspring of consanguineous marriages are also reportedly affected more than those of non-consanguineous marriages, indicating an autosomal recessive inheritance (20). The recent discovery and characterization of pre-Descemet’s layer has also improved the understanding of keratoconus and acute corneal hydrops, a rare but well-recognized complication of keratoconus (21–24).

Until the end of the 21st century, the management of keratoconus has been largely restricted to spectacles, rigid contact lens (CL) and keratoplasty (in advanced cases) for refractive and visual correction. Wollensak et al. (25) described a highly innovative and minimally invasive technique – corneal cross-linking (CXL) using the Dresden protocol – to halt the progression of keratoconus and reduce the need for keratoplasty. Since then, a variety of treatment protocols and techniques have been introduced to further optimize the clinical efficacy, efficiency, and safety of CXL. These include modifications such as accelerated CXL, transepithelial CXL, Epi-Flap CXL, pulsed UV light, and many others (26–34). Other surgical techniques, particularly intrastromal corneal ring segments (ICRS) and anterior lamellar keratoplasty (ALK), have been developed. In addition, there has also been a recent increased interest in the refractive surgical management of patients with keratoconus.

In this review, we aim to: (1) provide a comprehensive overview of the current therapeutic modalities of keratoconus; (2) propose a systematic and practical treatment algorithm; and (3) discuss the future directions of the management of keratoconus.

## 2. Important factors for consideration for treatment

The choice of treatment is contingent upon a combination of factors, including host factors (e.g., age, atopy, tolerance to CL, and visual requirement/expectations), clinical factors (e.g., severity and progression of keratoconus, location of the cone, corneal thickness, and presence of scarring or previous hydrops), and surgeons’ experience and expertise (35). A number of classifications have been proposed and used in the clinic to enable a more consistent grading of the severity of keratoconus, including the commonly used Amsler-Krumeich classification (36), Belin ABCD grading system (37), Keratoconus Severity Score (38), and several others (Table 1) (15, 39). The diagnosis of keratoconus has been well covered by a few recent excellent review articles (15, 35), and is beyond the scope of our article.

**TABLE 1:** Classification and grading of keratoconus based on Amsler-Krumeich classification and Belin ABCD grading system.

Amsler-Krumeich classification					
	**Eccentric CS**	Refraction*	**Mean central keratometry**	**CT**	**Scarring**
Stage 1	Yes	<5 D	<48 D	>400 μm	No
Stage 2	Yes	5–8 D	<53 D	>400 μm	No
Stage 3	Yes	8–10 D	>53 D	300–400 μm	No
Stage 4	Yes	Not measurable	>55 D	200 μm	Yes
**Belin ABCD grading system**					
	**A (ARC; 3 mm zone)**	**B (PRC; 3 mm zone)**	**C (thinnest pachymeter)**	**D (BDVA)**	**Scarring**
Stage 0	>7.25 mm (<46.5 D)	>5.90 mm (<57.25 D)	>490 μm	= 20/20	−
Stage 1	>7.05 mm (<48.0 D)	>5.70 mm (<59.25 D)	>450 μm	<20/20	−, +, ++
Stage 2	>6.35 mm (<53.0 D)	>5.15 mm (<65.5 D)	>400 μm	<20/40	−, +, ++
Stage 3	>6.15 mm (<55.0 D)	>4.95 mm (<68.5 D)	>300 μm	<20/100	−, +, ++
Stage 4	<6.15 mm (>55.0 D)	<4.95 mm (>68.5 D)	≤300 μm	<20/400	−, +, ++

CS, corneal steepening; CT, corneal thickness; ARC, anterior radius of curvature in the 3.0 mm zone centered on the thinnest location of cornea; PRC, posterior radius of curvature in the 3.0 mm zone centered on the thinnest location of cornea; BDVA, best-corrected-distance-visual-acuity. *Refraction refers to myopia and/or astigmatism.

The progression of keratoconus has been defined in several ways based on a combination of visual acuity, refraction, and tomographic/topographic indexes. Various parameters have been used in the literature and clinic to define progression (33, 40–42). These include:

- *Visual acuity:* subjective or objective decrease in vision by 1 Snellen line or more
- *Refraction:* increase in cylinder on manifest refraction by 1 D or more over 1 year
- *Keratometry:* increase in K2 (keratometry at the steepest meridian) or Kmax (maximum keratometry) by 1 D or more over 1 year
- *Corneal thickness:* progressive decrease (no definite quantitative value provided).

In 2015, the Global Delphi Panel of Keratoconus and Ectatic Disease has established a global consensus on the definition, concepts, diagnosis, clinical management, and surgical treatment of keratoconus and ectatic diseases (43). They have defined ectasia progression by a consistent change in at least two of the following parameters:

(1) Progressive steepening of the anterior corneal surface;
(2) Progressive steepening of the posterior corneal surface; and
(3) Progressive thinning and/or an increase in the rate of corneal thickness change from the periphery to the thinnest point.

**FIGURE 1:**
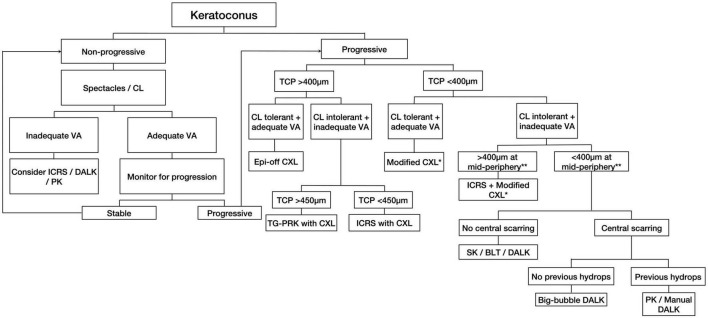
A proposed treatment algorithm for guiding the management of keratoconus. CL, contact lens; VA, visual acuity; ICRS, intracorneal ring segments; DALK, deep anterior lamellar keratoplasty; PK, penetrating keratoplasty; TCP, thinnest corneal pachymetry; CXL, corneal cross-linking; TG-PRK, topographic guided-photorefractive keratectomy; SK, stromal keratophakia; BLT, Bowman’s layer transplantation; DM, Descemet membrane. *Modified CXL includes transepithelial, iontophoresis-assisted, lenticule-assisted, CL-assisted, and adapted fluence CXL. **Corneal thickness at the mid-periphery/tunneling site for ICRS implantation.

More recently, the Belin ABCD Progression Display has also been introduced as an extension of the Belin ABCD grading system to detect and monitor the progression of keratoconus (44). This progression grading system considers both the anterior and the posterior corneal surfaces, which increases the sensitivity for detecting any early progression of the disease. Based on all the factors mentioned above, we propose a treatment algorithm as a practical (instead of prescriptive) guidance for managing keratoconus (Figure 1).

## 3. Conservative treatment

Eye rubbing, often in the context of ocular allergy, is often the main underlying predisposing factor for the development and progression of keratoconus (45–47). Therefore, avoidance of eye rubbing and a reasonable control of the underlying ocular allergy is crucial during the management of keratoconus. Studies have shown that a good control of ocular allergy can reduce the risk of disease progression, development of acute hydrops, and post-keratoplasty complications such as loose corneal graft sutures, persistent epithelial defect, and steroid-induced cataract (48–50). In recognition of the importance of patient education, a public awareness campaign, named the Violet June, was started in Brazil in 2018 to raise awareness of keratoconus and importance of avoiding eye rubbing in reducing the disease severity and its wider impact on the society (51). In addition, dry eye management is often required as the condition is common among patients with keratoconus who wear CL, irrespective of the types of CL used (52).

In the early stages of keratoconus, spectacles or soft CL may serve as a useful, first-line conservative treatment in providing satisfactory refractive and visual corrections. Refractive errors are commonly measured with manifest clinical refraction but may be objectively aided by ocular wavefront analysis (35). However, as the disease often affects the eyes asymmetrically, many affected individuals remain asymptomatic until one eye is significantly affected or both eyes are considerably affected, rendering the above treatment options unsatisfactory. Occasionally, early detection of the disease may occur by chance during routine eye screening by optometrists in the community and/or ophthalmologists.

The use of CL in cases with keratoconus was first introduced by Fick (53). Since then, significant advancement has been made in the designs of CL used for keratoconus. In general, the use of CL in keratoconus depends on the stage of keratoconus, the location of the cone, and patient’s variable tolerance to CL. Soft toric CL offers the advantage of increased comfort. However, they cannot correct higher order aberrations (HOAs) and are best suited for early keratoconus (54). When used in advanced stages, conflicting results have been reported, with some authors finding it difficult to fit these CLs (55) while others report good results (56, 57). Rigid gas permeable (RGP) CL is the preferred option for keratoconic corneas. Customized RGP CL has been introduced to address the challenges in the fitting of traditional RGP CL (58). Studies have shown good RGP CL fitting in stage 2 and stage 3 keratoconus (56), though the vision-related quality of life (VR-QoL) is reduced when they were used for corneas with keratometry values exceeding 52 D (58).

When RGP CL wear is not tolerated, several other options are available to improve CL tolerance in patients with keratoconus. Hybrid CLs consist of a central rigid zone with a peripheral soft skirt, which provides better comfort and potentially better visual acuity than those using RGP CLs (59). They are better fitted for patients with stage 1 and stage 2 keratoconus and have been shown to improve their VR-QoL (59). However, hybrid CLs are associated with potential increased risks of giant papillary conjunctivitis, corneal edema, and vascularization (60, 61). Mini-scleral and scleral CLs are strong alternatives to RGP corneal CLs in patients with very steep or irregular corneas seen in advanced keratoconus (62). These CLs have a larger diameter and rest on the sclera without touching the limbus and the cornea, building a fluid reservoir between the posterior surface of the CL and the cornea that helps in evening out the irregularities and maintaining epithelial health. The prosthetic replacement of ocular surface ecosystem (PROSE) CL has been shown to be highly effective in patients with excessive HOAs (63). They are also particularly beneficial in patients with keratoconus having co-existing ocular surface disease (64). Piggyback CL, consisting of a soft bandage CL and an overlying RGP CL, also serves as another valuable option for vision correction in patients with keratoconus who are intolerant to RGP CL and scleral lens (65).

## 4. Surgical treatment

### 4.1. Corneal cross-linking

Corneal cross-linking (CXL) refers to the formation of covalent bonds between collagen molecules which results in biomechanical tissue strengthening (66). Physiological age-related collagen cross-linking has been shown to occur naturally whereby the diameter of the corneal collagen fibrils increases by up to 4.5% in an individual’s lifetime (67). Researchers noted that diabetic patients rarely developed keratoconus due to glycosylation-related collagen cross-linking and developed a technique for chemical CXL in biomechanically weaker corneas such as in keratoconus (68). The University of Dresden described the process of CXL using riboflavin and ultraviolet (UV-A) as a treatment modality for keratoconus. A complex photochemical reaction consisting of aerobic and anaerobic phases leads to the formation crosslinks between collagen molecules (69). Studies have also shown that CXL strengthens the cornea via cross-linking of the collagen molecules and non-collagen molecules (70, 71); therefore, the term “corneal cross-linking,” instead of “corneal collagen cross-linking,” is more commonly used in current practice.

**FIGURE 2:**
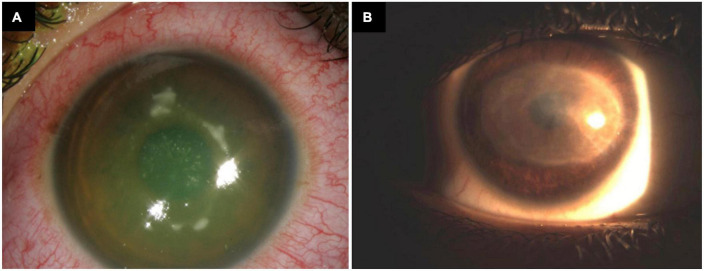
Infectious keratitis following epithelium-off corneal cross-linking for progressive keratoconus. **(A)** Active infection. **(B)** Resolved infection with a visual debilitating corneal scar.

Since the original CXL technique was described, several modifications have been described and used to reduce the time and increase the efficiency of CXL in stabilizing keratoconus. The long-term efficacy and safety of CXL have also been well-established (71, 72), though postoperative complications such as infectious keratitis, reactivation of herpes simplex keratitis, acute hydrops, endothelial damage, and corneal haze/scar may occur (Figure 2) (73, 74).

#### 4.1.1. Epithelium-off techniques

##### 4.1.1.1. Dresden protocol (conventional protocol)

The Dresden protocol or the conventional protocol of CXL (C-CXL) was originally described by Wollensak et al. (25). Since then, several prospective and retrospective studies have established the efficacy of C-CXL in halting the progression of keratoconus (42, 75, 76). The technique involves epithelial debridement in the central 8–9 mm zone followed by soaking the cornea in 0.1% riboflavin solution for 30 min before irradiating the cornea with 370 nm UV-A light (3 mW/cm^2^ for 30 min) to achieve a surface dose of 5.4 J/cm^2^. This technique is currently considered as the standard for CXL and is often performed in outpatient settings. The corneal epithelium can be removed using alcohol (33, 77, 78), hockey knife (79), Amoils brush (80), or transepithelial phototherapeutic keratectomy (PTK) (81, 82). Removal of the hydrophobic corneal epithelium facilitates adequate riboflavin penetration and imbibition into the stroma, allowing for effective UV-A induced photochemical reactions and subsequent CXL. The degree of riboflavin penetration affects the depth of UV-A radiation absorption thereby affecting the extent of CXL. Immediate post-CXL corneal haze and/or apoptosis of keratocytes in the anterior and middle stroma, which is often observed as a demarcation line in the anterior segment optical coherence tomography (AS-OCT), may serve a surrogate marker for the depth of CXL (83, 84).

Studies on corneal biomechanics have proven the stiffening effect of CXL on animal as well as human corneas (85, 86). C-CXL has also been shown to improve the corneal curvature reducing the corneal steepening and improving visual acuity (87, 88). Corneal thinning is noted up to 3 months post-surgery after C-CXL, which slowly recovers by 1 year (89–91). However, Kim et al. (92) demonstrated that even after 5 years, there was a statistically significant reduction in corneal thickness as compared to the pre-CXL values. In general, CXL achieves better efficacy when performed in early keratoconus as compared to advanced keratoconus (33, 93). Innovative approach such as Epi-Flap CXL has also been described, which is shown to be associated with less postoperative pain and anterior stromal haze when compared to conventional epithelium-off CXL (34).

##### 4.1.1.2. Accelerated protocol

The concept of accelerated CXL (A-CXL) is based on the Bunsen-Roscoe law of photochemical reciprocity, which states that the total energy remains unchanged if the irradiation duration is reduced with a corresponding increase in the intensity of irradiation (69). The main advantages of A-CXL include reduced treatment duration and a possible reduced risk of infection (94). Several protocols for A-CXL have been described, including the use of 9 mW/cm^2^ for 10 min, 18 mW/cm^2^ for 5 min, and 30 mW/cm^2^ for 3 min, all of which result in a surface dose of 5.4 J/cm^2^ (33, 95, 96). Larger dose of radiation such as 7.2 J/cm^2^ (with 30 mW/cm^2^ radiation for 3 min) has been described, though it may lead to increased risk of corneal haze (97). Laboratory studies analyzing the corneal stiffening following CXL have shown comparable efficacy of C-CXL with A-CXL (98, 99). A large *ex vivo* study showed that the Bunsen-Roscoe law of reciprocity is valid only up to irradiation intensities of 40–50 mW/cm^2^ and for more than 2 min. At higher intensities and shorter duration, the biomechanical stiffening can rapidly decrease (100).

Many large clinical studies have demonstrated the clinical efficacy and safety of A-CXL in halting progressive keratoconus in adult patients (33, 101–103). The potential benefit and safety of A-CXL has also recently been substantiated in pediatric patients with progressive keratoconus. Larkin et al. (104) recently conducted the KERALINK study, a phase 3 clinical trial which included 60 patients aged 10–16 years with progressive keratoconus. It demonstrated a significantly beneficial effect of A-CXL [using continuous UV light with 10 mW/cm^2^ over 9 min (total energy of 5.4 J/cm^2^)] in halting the progression of pediatric keratoconus when compared to those receiving standard care (which included refraction testing with provision of glasses and/or CL fitting). CXL was offered to the standard care group if there was confirmed disease progression but this was not provided earlier than 9 months after randomization. The reported follow-up period was 18 months.

A meta-analysis of 11 studies evaluating the efficacy and safety between A-CXL and C-CXL demonstrated similar postoperative outcomes, in terms of mean keratometry (K_mean_), uncorrected-distance-visual-acuity (UDVA), and corrected-distance-visual-acuity (CDVA) (105). However, A-CXL was shown to achieve less reduction in maximum keratometry (K_max_) but with less impact on corneal thickness and endothelial cell density when compared to C-CXL. The observed difference in the effect (in K_max_) may be attributed to less energy penetration into the cornea hence less corneal biomechanical stiffening in the A-CXL group, evidenced by shallower corneal demarcation line in A-CXL when compared to C-CXL (69, 84). Touboul et al. (106) reported a corneal demarcation line at a depth of 100–150 μm using a 30 mW/cm^2^ irradiance protocol for 3 min. This is possibly due to the shorter riboflavin soaking time of 10 min in their study. Studies with riboflavin soakage for 15 min have reported the demarcation line to be deeper than 200 μm (26, 107). Kymionis et al. (108) found a demarcation line at a mean of 288–290 μm, using a protocol of 9 mW/cm^2^ for 10 min. A study comparing the depth of demarcation lines in two groups of patients undergoing A-CXL with 18 mW/cm^2^ for 5 min and different riboflavin soakage times (20 and 30 min) found that it was deeper in the group with longer soakage time before UV-A irradiation (109).

Favorable outcomes have also been reported in terms of keratometric flattening and improvement in visual acuity using A-CXL (110, 111). Razmjoo et al. (112) reported comparable improvement in visual acuity and refractive astigmatism after A-CXL and C-CXL. The photochemical reaction during CXL is known to be oxygen-dependent (113). Accelerated protocols with higher fluence raised concerns about oxygen depletion during CXL, resulting in a reduced stiffening effect. This led to the concept of using pulsed light during irradiation instead of continuous light. Pulsing the light allows more oxygen availability and more singlet oxygen release for CXL (30). Comparative studies of continuous light A-CXL and pulsed light A-CXL have revealed a deeper demarcation line when pulsed light was used, with the latter achieving a better efficacy (30, 114). Studies have shown that pulsed light A-CXL is effective in halting the progression of keratoconus up to 2 years after the procedure (115, 116).

#### 4.1.2. Epithelium-on techniques

##### 4.1.2.1. Transepithelial CXL

CXL without epithelial debridement has been described to reduce the risk of infectious keratitis and improve patient comfort after the procedure (74, 117–119). However, riboflavin, a hydrophilic substance with a high molecular weight, does not penetrate through an intact hydrophobic epithelium easily (120). This issue is overcome by using permeability enhancers like ethylene-diamine-tetra-acetic acid (EDTA), benzalkonium chloride (BAC), trometamol, and gentamicin (121, 122). The riboflavin-soaked epithelium also absorbs UV radiation resulting in an attenuated effect of CXL (123). Studies have shown that transepithelial CXL is less effective in halting progression in keratoconus than C-CXL (124). Bottós et al. (125) suggested that the reduced effect of transepithelial CXL is due to inadequate stromal concentration of riboflavin rather than reduced UV transmittance. Reduced oxygen diffusion into the stroma due to an intact epithelium also attenuates the effect of CXL (126). It has been estimated that corneal biomechanics improves by 64% following transepithelial CXL as compared to 320% after C-CXL (127). The demarcation line after transepithelial CXL is also reportedly shallower than C-CXL (127). The use of pulsed treatment (1 s on, 1 s off) to improve oxygen concentration has improved the results at 1-year follow-up (128). A study by Caporossi et al. (28) reported that the UDVA and CDVA improved up to 3–6 months then gradually returned to the preoperative level. Keratoconus was noted to be stable up to 1 year, however there was a subsequent worsening noted at 2-year follow-up. They used riboflavin with dextran and EDTA, and trometamol as the permeability enhancers. Another study done using riboflavin with BAC 0.01% reported a progression of keratoconus in 46% eyes at 1-year follow-up (129). Other studies have concluded that, although limited, there is a definite favorable effect of transepithelial CXL (130, 131). A recent Cochrane review of 11 studies reported that transepithelial CXL and epithelium-off CXL confer similar efficacy on keratoconus stabilization (132). Another approach recently described by Mazzotta et al. (133) is to increase the fluence to 7 J/cm^2^ and use pulsed UV light. This approach has been termed as Transepithelial Enhanced Fluence Pulsed M Accelerated Crosslinking (EFPL-M-CXL).

##### 4.1.2.2. Iontophoresis-assisted CXL

Riboflavin is a negatively charged, small molecule with a molecular weight of 376.4 g/mol. Penetration of riboflavin into the corneal stroma (via an intact layer of epithelium) can be enhanced using iontophoresis, a non-invasive technique employed to facilitate the movement of ionized molecules (134). In this technique, the active electrode is placed over the cornea with a suction ring, while the passive electrode is placed on the cervical vertebrae or on the patient’s forehead. The annular suction ring on the cornea is then rinsed with 0.1% riboflavin in distilled water until the grid is submerged. A small current of 1 mA is then applied for 5 min. Once adequate stromal soakage by riboflavin is confirmed through slit-lamp examination, the cornea is then irradiated with UV-A light (135, 136).

Studies have shown that iontophoresis-assisted CXL (I-CXL) was effective in halting progression at 1-year follow-up (135, 137). The depth of the corneal demarcation line has been reported to be at 210 μm with no significant corneal haze (135). Studies comparing C-CXL and I-CXL in early keratoconus have shown comparable effects in stabilizing progressive keratoconus (138, 139). Another study reported that at a 2-year follow-up, I-CXL could halt keratoconus, albeit less efficiently than C-CXL (80 vs. 92.5%). They reported a demarcation line at a depth of 216 μm observed in 35% of cases. The failure rate reported was 20% with I-CXL as compared to 7.5% with conventional CXL (140). Similar to transepithelial CXL, the efficacy of I-CXL is reduced by the presence of intact corneal epithelium due to decreased oxygen diffusion. Secondly, the riboflavin-soaked epithelium is likely to reduce the UV transmittance. A study by Mastropasqua et al. (141) showed that riboflavin saturation achieved in I-CXL was deeper than transepithelial approach, but shallower than the conventional epithelium-off CXL approach.

Some potential advantages do exist with I-CXL. Studies have shown that contrast sensitivity function was transiently better after I-CXL than C-CXL during early postoperative period (first 3 days), which is likely attributed to the inflammatory effect during the wound healing process following epithelial debridement in C-CXL (142, 143). However, the significant difference observed between the two groups was normalized by 1 week postoperative. In addition to the reduction of the riboflavin soakage time to 5 min, it also offers the advantages of intact epithelium such as reduced pain and lower chances of infectious keratitis (118, 144).

#### 4.1.3. Modified techniques for thin corneas

##### 4.1.3.1. Lenticule-assisted CXL

Sachdev et al. (145) first described an innovative lenticule-assisted CXL technique in three patients where tailored stromal expansion was used for corneas <400 μm, utilizing lenticules extracted from patients undergoing small incision lenticule extraction (SMILE). Following epithelial debridement, a stromal lenticule of appropriate thickness derived from a patient undergoing SMILE for myopic correction was placed on the cornea to be cross-linked. The lenticule was centered over the apex of the cone. Riboflavin 0.1% was instilled every 5 min for 30 min, and UV-A radiation was given as in the Dresden protocol. The effectiveness and safety of this technique was observed throughout their 6-month study, evidenced by the keratometric stability, minimal/no effect on the endothelial cell density, and a corneal demarcation line of 280–300 μm (suggesting that the posterior stroma and endothelium is spared). The thickness of the lenticule can also be customized according to the corneal thickness of the treated eye. Further modification was described by Cagini et al. (146) where they prepared the lenticule using femtosecond laser (FSL) on donor corneas and standardized the lenticule thickness to 100 μm. Their study included 10 eyes of eight patients, and the demarcation line at 1 month was comparable to C-CXL technique. Further studies are needed to determine the efficacy and safety of this technique.

##### 4.1.3.2. Contact lens-assisted CXL

The technique of CL-assisted CXL (CL-CXL) was first described by Jacob et al. (147) in 2014 in their pilot study of 14 eyes with thin cornea. In this technique, the cornea was soaked with riboflavin for 30 min after epithelial debridement. A CL was also soaked in riboflavin solution simultaneously for 30 min. This riboflavin-soaked CL was then placed on the cornea before UV irradiation was started. Intra-operative pachymetry was done to confirm the thickness to be >400 μm. UV-A irradiation was then given as in the Dresden protocol. Placing a CL on the thin cornea increases the available thickness of the cornea by approximately 100 μm. Usually a CL with negligible power is chosen. The hydrophilicity of the CL is another factor to be considered. A more hydrophilic CL absorbs more riboflavin and hence more UV-A radiation, thereby reducing the transmission of UV-A to the stroma and affecting the effect of CXL (148). Wollensak et al. (148) compared the biomechanical efficacy of CL-CXL with C-CXL in porcine eyes and found that CL-CXL was about one-third less efficacious than C-CXL. Using Brillouin microscopy, Zhang et al. (149) reported that the localized corneal stiffening effect after CL-CXL was up to 70% that after standard CXL. Both groups showed a higher cross-linking effect in the anterior cornea than in the middle and posterior cornea. Knyazer et al. (150) performed CL-CXL using the accelerated protocol on 24 eyes with keratoconus. Progression was halted in 80% of cases with no evidence of damage to the corneal endothelium.

##### 4.1.3.3. Adapted fluence CXL

Approaches like CL-CXL and hypotonic riboflavin have their drawbacks of having limited biomechanical strengthening (151). Kling and Hafezi (152) suggested that the rate-limiting factor in CXL is the irradiation time rather than the UV fluence or irradiance. If the fluence or irradiance is varied, it would be possible to perform CXL in corneas thinner than 400 μm since the threshold for endothelial cell toxicity would not be surpassed. Based on this theory, they proposed an algorithm for CXL in thin corneas where the fluence was adapted according to the pre-irradiation thickness. In their retrospective analysis of 39 eyes, they found a significant correlation between the irradiation time and the demarcation line depth. At 1-year follow-up, progression of keratoconus was halted in 90% of the patients and none of the patients developed endothelial decompensation (153). Further studies are needed to assess the long-term stability of such cases.

### 4.2. Intracorneal ring segments

In 1949, Dr. Barraquer explored the placement of an intracorneal device to correct a patient’s myopia, though this approach has become obsolete for this indication (154). It was only in 2000 that Colin et al. (155) reported the ability of implanted devices, now known as ICRS, to reduce corneal steepening in keratoconic eyes with some improvement in UDVA, CDVA, and tolerance to CL wear.

**FIGURE 3:**
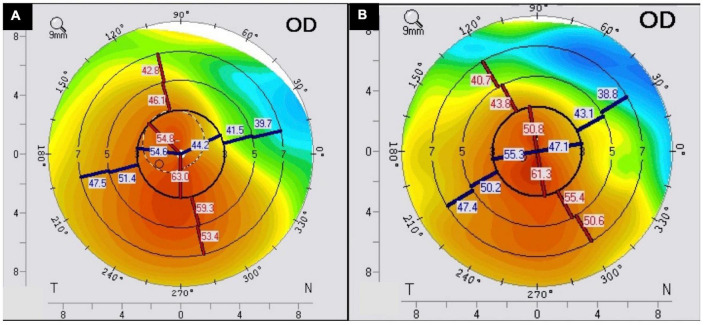
A case of intracorneal ring segments (ICRS) implantation for treating stable moderate keratoconus. The **(A)** preoperative and **(B)** postoperative corneal topography demonstrates an improvement in the regularity of the cornea following ICRS.

This technique works on the principle that adding tissues at the corneal periphery confers a centrally flattening effect (known as the arc shortening effect) (154). Patients with keratoconus whose vision can no longer be corrected adequately with spectacles or those intolerant to CL wear may benefit from this minimally invasive and reversible procedure. Other less common indications for ICRS include irregular astigmatism post-penetrating keratoplasty (PK) or deep anterior lamellar keratoplasty (DALK), post excimer laser corneal ectasia, astigmatism post radial keratotomy, and pellucid marginal degeneration. Whilst PK and DALK can be the next surgical step in keratoconus patients with visual impairment not correctable by spectacles or CLs, ICRS serves as a useful surgery to bridge the gap, delay or even eliminate the need for keratoplasty (Figure 3). There has also been some debate on whether or not ICRS prevents disease progression, with some studies supporting its long-term stabilizing effect whilst the others did not (156–160).

Since the first human ICRS implantation report in the early nineties (161), various types of polymethyl methacrylate (PMMA)-based ICRS have now become widely available globally (Table 2). When considering ICRS implantation in keratoconus patients, ideally the cornea should be without scarring, though recent evidence suggests that corneas with mild apical haze may still benefit from ICRS (162).

**TABLE 2:** A summary of different types of intracorneal segment rings.

Type/company	Number of segments	Cross section	Size (μm)	Diameters (Int/Ext)	Arc length (degree)	Optical zones (mm)
Keraring, Mediphacos, Brazil	2	Triangle	150–350 (50 steps)	5/6	90, 120, 150, 160, 210, 340, 355	5, 5.5, 6
Ferrara, Ferrara Ophthalmics, Brazil	2	Triangle	150–350 (50 steps)	4.4/5.6	90, 100, 110, 120, 130, 140, 150, 160, 170, 180, 190, 210, 320	5, 6
Intacs, Addition Technology, Lombard, IL, USA	2	Hexagon	250–450 (50 steps)	6.7/8.1	150, 210	7
Intacs SK, Addition Technology, Lombard, IL, USA	2	Oval	210–500 (50 steps)	6/7	90, 130, 150	6
Corneal Ring, VisionTech, Brazil	2	Fusiform	150–350	4.7/6	155–220	5, 6
MyoRing, Dioptex GmbH, Austria	1	Triangle	200–320	5–8	360	5–8
KentacX, Plus (Imperial Medical Technologies Europe GmBH)	2	Triangle with rounded corners	100–450 (25 steps)	4.5–5.7	45, 90, 120, 160, 210, 320, 355	5.4–5.6
Bisantis (Opticon 2000 SpA y Soleko SpA)	Up to 4	Oval	150	3.5	80	3.5, 4, 4.5

Pre-operatively, one may consider an implantation checklist including but not exhaustive of the following: (1) CDVA of 20/30 or worse; (2) CL intolerance; (3) corneal thickness of more than 400 μm at the site of the tunnel; (4) steep keratometry less than 62 D; (5) adequate stromal bed around the rings (thickness of the ring cannot exceed half that of the corneal thickness); and (6) patient’s acceptance of a moderate and slow improvement in CDVA as management of the patient’s expectation is key (163, 164). Though pediatric patients have been implanted with ICRS, it is more commonly accepted to be placed in patients 18 years of age or older (165, 166).

So far, ICRS has been shown to be an effective method in managing keratoconus, with many recent studies reporting a significant improvement in UDVA, CDVA, refraction, and keratometric readings (Table 3) (159, 167–175). In a sizeable multi-centered study of 611 eyes with varying degree of keratoconus, Vega-Estrada et al. (176) observed a significant improvement in UDVA in all cases of keratoconus and CDVA in the majority of cases (except for mild keratoconus with 0.1 logMAR vision or better). This suggests that ICRS is most beneficial in grade I to III keratoconus. In addition to symmetric ICRS implantation, there have recently been some studies reporting the potential benefit of asymmetric ICRS implantation for keratoconus. However, the evidence is largely limited to short follow-up and small sample size (177, 178). Furthermore, novel progressive thickness ICRS (i.e., one end of the ring is thicker than the other end) has been successfully used to treat keratoconic eyes with non-uniform irregularities, resulting in a progressive corneal flattening effect (179, 180). Recently, Alfonso-Bartolozzi et al. (181) also reported using FSL-assisted, Ferrara-type ICRS to effectively correct astigmatism (≥3.00 D) following DALK.

**TABLE 3:** A summary of the recent studies evaluating the effectiveness and stability of intracorneal ring segment (ICRS) for keratoconus.

References	Number of eyes	Intervention	Visual outcomes	K change (D)	Stability	Follow-up duration
Costa et al. (159)	124	Ferrara	UDVA (logMAR): 0.91 ± 0.36 (preop) to 0.46 ± 0.32 (postop), CDVA (logMAR): 0.40 ± 0.27 (preop) to 0.22 ± 0.20 (postop)	K_max_: −1.8	Stability: 90.3%	5 years
Peris-Martínez et al. (171)	61	Ferrara	UDVA (logMAR): 0.89 ± 0.52 (preop) to 0.44 ± 0.34 (postop)	K_max_: −4.0	Stable (8.2% extrusion)	2 years
de Araujo et al. (169)	34	Ferrara	CDVA (logMAR): 0.32 ± 0.19 (preop) to 0.46 ± 0.27 (postop)	K_max_: −3.1	Stability: 74%	5 years
Warrak et al. (175)	932	Intacs	Both UDVA and CDVA improved significantly	K_max_: −3.8	Stability: 97.3%, extrusion rate: 0.4%	3 years
Prisant et al. (172)	82	Keraring	UDVA (logMAR): 0.82 (preop) to 0.46 (postop), CDVA (logMAR): 0.31 (preop) to 0.21 (postop), Mean change in SE: 0.8 D	K_max_: −3.3	Stable (but short follow-up)	3 months
Abdellah and Ammar, (168)	38	Keraring 355	UDVA (logMAR): 0.93 ± 0.21 (preop) to 0.63 ± 0.21 (postop), Mean change in SE: 2.9 D	K_max_: −3.4	High rate of extrusion (31.5%) and instability	3 years
Kang et al. (170)	30	Intacs	UDVA (logMAR): 0.86 ± 0.46 (preop) to 0.74 ± 0.37 (postop), CDVA (logMAR): 0.52 ± 0.30 (preop) to 0.40 ± 0.30 (postop), Mean change in SE: 1.1 D	K_mean_: −2.9	Only stable cases were included in this study.	5 years
Torquetti et al. (174)	138	Ferrara 320	UDVA (logMAR): 1.1 (preop) to 0.3 (postop), CDVA (logMAR): 0.7 (preop) to 0.3 (postop), Mean change in SE: 3.7 D	K_mean_: −5.5	Stable (but short follow-up)	6 months
Abd Elaziz et al. (167)	30	Keraring 355	CDVA (decimal): 0.22 ± 0.17 (preop) to 0.49 ± 0.22 (postop), Mean change in SE: 7.6 D	K_max_: −8.6	Stability: 90%, migration/extrusion: 10%	6 months
Sandes et al. (173)	58	Ferrara 140	CDVA (logMAR): 0.5 ± 0.2 (preop) to 0.3 ± 0.21 (postop)	K_mean_: −2.5	Good stability with 88% retainment rate	17 months

UDVA, uncorrected-distance-visual-acuity; CDVA, corrected-distance-visual-acuity.

There are a few contraindications to ICRS implantation, including advanced keratoconus with more than 65 D keratometry (62–65 D being a gray area), corneal opacity, severe atopic disease, eye rubbing, immunological diseases, corneal dystrophy, pregnancy, and breastfeeding (164). Patients with a history of herpetic eye disease as well as those on isotretinoin, amiodarone, and sumatriptan are not favorable candidates. Wound healing may be affected in patients with diabetes and severe atopic disease. It is also preferable to avoid patients with large pupils, as the chances of inducing HOAs are high. Corneal topography, aberrometry and assessment of the corneal biomechanics are helpful.

**FIGURE 4:**
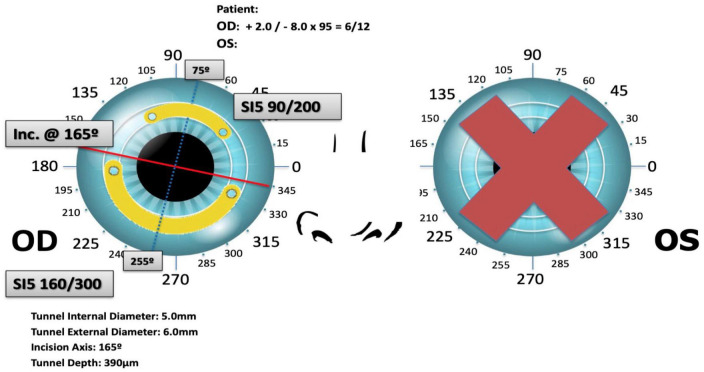
Surgical planning of Keraring intracorneal ring segments implantation based on preoperative refraction and tomography.

Different nomograms exist for each type of ring, with the size and position usually planned by the manufacturers based on refraction and tomography (Figure 4). For example, for Kerarings, one must send the refraction and tomography, including the four refractive maps, four topometric maps, one color map, and a corneal Zernike analysis to the 6th order over a maximum 6 mm pupil. It is thought that refractive and topographic cylinders should be within 15° of each other for a better result (look at K1 and refractive cylinder axis) (164). Usually, two segments are placed, with thinner segments for mild cases. ICRS may be placed by manual dissection or FSL dissection, where a tunnel or channel is created in the deep corneal stroma. Studies have shown that both FSL and manual dissection techniques result in similar improvement in visual, refractive and topographic outcomes, though the FSL technique has a better safety profile with a lower complication rate (164, 182).

**FIGURE 5:**
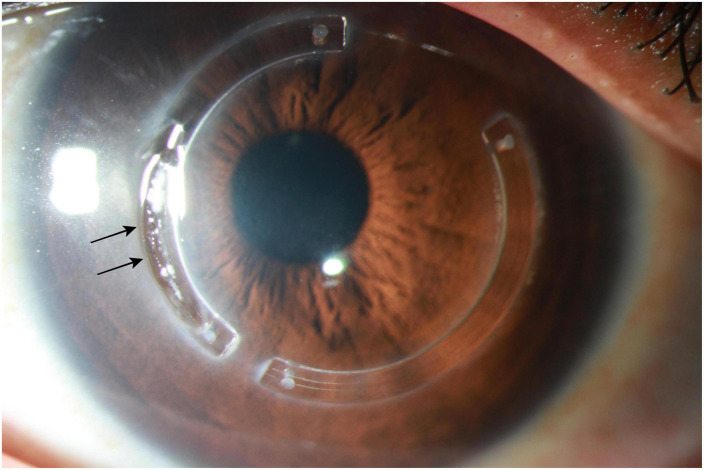
Slit-lamp photograph demonstrating an extrusion of intracorneal ring segments (black arrows), which is a well-recognized complication.

Complications of ICRS include ring exposure or extrusion (Figure 5), broken ring segments, segment migration, recurrent erosion, corneal melt, perforation, vascularization, and infectious keratitis (183–185). Coskunseven et al. (186) reported 1.3% of cases had segment migration, 0.2% cases of corneal melting, and 0.1% case of mild infection, with an overall complication rate of 5.7% (49 out of 850 eyes). Long-term stability has been shown in adults but less so in the pediatric population, associated with a higher chance of progression (156, 187–192). Variable thickness ICRS and Long Arc Length ICRS are both being explored, though their effectiveness is less in advanced keratoconus, which is similarly observed in ICRS in general (172, 178). The explantation rate due to complications or poor visual outcomes (including halo and glare) is considered relatively low.

To improve the biocompatibility and biointegration of ICRS within the host cornea, Jacob et al. (193) described a novel technique of corneal allogenic intrastromal ring segments (CAIRS) combined with CXL to manage patients with stage I to IV keratoconus. A customized double-bladed trephine with outer diameter 7.5 mm and inner diameter 6.7 mm was used to cut the donor corneal rims into ring-like segments. The lenticules were crosslinked and cut into two halves akin to INTACS segments and inserted intrastromally at 50% depth of the thinnest pachymetry at 7 mm zone. Their study of 24 eyes (20 patients) showed a significant improvement in UDVA, CDVA, and steepest keratometry values at 18 months after the procedure. None of the patients had displaced or extruded segments. Transient dehydration of the CAIRS before insertion has also been described to reduce the surgical learning curve and aid insertion of thicker segments for more severe keratoconus (where the cornea is very thin and insertion of thick segments are technically challenging) (194). Another similar study was conducted by Jafarinasab and Hadi (195) where they created ring-like stromal segments from the anterior lamellae left after preparing DSAEK lenticules. The authors reported an improvement in CDVA without a significant difference in refractive or topographic astigmatism.

In summary, ICRS, based on either synthetic material or human donor cornea, serves a safe, effective, reversible, and adjustable surgical option for managing keratoconus, with variable outcomes dependent on the differing experiences between surgeons and units.

### 4.3. Combined CXL-keratorefractive surgeries

It is well known that keratorefractive surgeries may result in postoperative corneal ectasia, particularly in patients with underlying undetected mild keratoconus or forme fruste keratoconus (196). Therefore, keratorefractive surgery is traditionally contraindicated for eyes with keratoconus. However, with the growing experience in CXL and refractive surgery over the past decade, more clinicians are combining these two procedures, simultaneously (on the same day) or sequentially, to visually and refractively rehabilitate patients with keratoconus (35, 197, 198). “CXL plus” is a term coined in 2011 to refer to any CXL combined with refractive surgery to treat corneal ectasia (199). CXL and ICRS, either coupled or decoupled, have shown a significant improvement in keratometry (200). The results of this can be enhanced further with refractive surgery such as topography-guided photorefractive keratectomy (TG-PRK) (201).

Multiple combinations exist for CXL plus, including PRK, transepithelial PRK (t-PRK), TG-PRK, transepithelial TG-PRK, and wavefront-guided PRK (WG-PRK). Combined with varying CXL protocols and intra- and inter-person variability (as part of the nature of the disease), it is understandable why optimum parameters are difficult to determine. However, there is cumulative evidence in the literature demonstrating the long-term effectiveness and safety of various CXL plus procedures, including CXL + ICRS (189, 202–204), CXL + PRK/PTK (205–212), and CXL + PRK/PTK ± phakic IOL implantation (Table 4) (213–217).

**TABLE 4:** A summary of some of the recent studies examining the effectiveness and safety of corneal cross-linking (CXL) plus procedures for managing keratoconus (this is not an exhaustive list).

References	Number of eyes	Intervention	Visual outcome	Refractive/K changes	Complications	F/U (months)
***CXL* + *ICRS***						
Hersh et al. (203)	198	CXL + ICRS (concurrent vs. sequential)	Concurrent: UDVA improved 0.17 logMAR, CDVA improved 0.09 logMAR, Sequential: UDVA improved 0.24 logMAR, CDVA improved 0.14 logMAR	K_max_: −2.2 D (concurrent) vs. −2.7 (sequential)	2 (1%) cases of microbial keratitis (1 in each group)	6
Renesto Ada et al. (204)	39	Intacs vs. CXL then Intacs	Intacs: UDVA improved 0.16 logMAR, CDVA improved 0.13 logMAR, CXL/Intacs: UDVA improved 0.33 logMAR, CDVA improved 0.22 logMAR	No difference between groups for all K values	Nil	24
Kilic et al. (189)	131	Same-day Intacs + epi-on CXL	UDVA improved 0.26 ± 0.16 logMAR, CDVA improved 0.24 ± 0.16 logMAR	Kmean: −4.5 D	Nil	1–25
Coskunseven et al. (202)	48	CXL then ICRS vs. ICRS then CXL	CXL/ICRS: UDVA improved 0.18 decimal, CDVA improved 0.17 decimal, ICRS/CXL: UCVA improved 0.21 decimal, BCVA improved 0.33 decimal	CXL/ICRS: K_mean_: −4.2 D ICRS/CXL: K_mean_: −4.0 D	8 eyes with stromal edema (resolved by 3 months)	13
***CXL* + *PRK/PTK***						
Iqbal et al. (207)	125	PRK + CXL (Athens) vs. CXL alone	CXL: UDVA improved 0.54 logMAR, PRK + CXL: SE reduced 2.3 D, UDVA improved 0.68 logMAR	CXL: K_mean_: −2.1 D, SE reduced 2.3 D, PRK + CXL: K_mean_: −1.4 D, SE reduced 2.3 D	CXL: 12.1% haze, 1.7% stromal scar CXL + PRK: 5.9% haze, 1.3% stromal scar	24
Ohana et al. (212)	98	Athens	UDVA improved 1.23 logMAR, CDVA no improvement	K_mean_: −4.0 D	5% significant haze	12–36
Nattis et al. (211)	62	CXL then TG-PRK	Ref: UDVA 20/100 to 20/60 CDVA 20/50 to 20/30	Ref: K_mean_: −0.4 D, Topo: no change	Nil	6
Gore et al. (206)	47	Athens	CDVA improved 0.13 logMAR	K1: −1.1 D, K2: −5.4 D	Microbial keratitis (2%)	24
Kontadakis et al. (210)	60	TG-tPRK + CXL vs. CXL alone	PRK + CXL: CDVA improved 0.17 logMAR, CXL: CDVA improved 0.09 logMAR	Significant improvement in K1 and K2 in the PRK + CXL group	Nil	39
Kanellopoulos and Asimellis (209)	231	Athens	UDVA improved 0.38 ± 0.31 logMAR, CDVA improved 0.20 ± 0.21 logMAR	K1: −3.4 D	Nil	36
Alessio et al. (205)	34	PRK + CXL vs. CXL alone	PRK + CXL: UDVA improved 0.44 logMAR, CDVA improve 0.03 logMAR, CXL: UDVA improved 0.07 logMAR, CDVA improved 0.02 logMAR	Significant improvement in SE, K1, K2, and Kmax in PRK + CXL group but not in CXL group	Nil	24
Kanellopoulos (208)	325	Athens (simultaneous vs. sequential)	Sequential: UDVA improved 0.41 logMAR, CDVA improved 0.25 logMAR Simultaneous: UDVA improved 0.66 logMAR, CDVA improved 0.28 logMAR	Sequential: K_mean_: −2.8 ± 1.3 D Simultaneous: K_mean_: −3.5 ± 1.3 D	Stromal haze: 13.4% (sequential) vs. 1% (simultaneous)	24–68
***CXL plus (3 or more procedures)***						
Shetty et al. (217)	48	ICRS followed by CXL + TG-tPTK	UDVA improved 0.53 logMAR, CDVA improved 0.11 logMAR	SE: 4.6 D, Cyl: 1.7 D	2% lost 1 line of CDVA	12
Rocha et al. (216)	55	Simultaneous ICRS + PTK + CXL	UDVA Improved 0.39 logMAR, CDVA improved 0.08 logMAR	Cyl: −2.1 D	1 eye (2%) lost >3 lines CDVA (haze)	6
Assaf and Kotb (213)	22	Sequential Athens protocol then PIOL	UDVA improved 0.87 logMAR, CDVA improved 0.34 logMAR	K_mean_ reduced −1.8 D	Nil	6–14
Coskunseven et al. (214)	16	ICRS then TG-tPRK then CXL	UDVA improved 0.89 logMAR, CDVA improved 0.62 logMAR	SE: 4.7 D, K1: −3.1 D, K2: −8.7 D	Nil	6
Kremer et al. (215)	45	Sequential Intacs then same-day PRK + CXL	UDVA improved 0.35 decimal, CDVA improved 0.19 decimal	K_max_: −4.3 D	11.1% mild haze	12

K, keratometry; F/U, follow-up; TG, topography-guided; PRK/PTK, photorefractive/phototherapeutic keratectomy; tPTK, transepithelial phototherapeutic keratectomy; ICRS, intrastromal corneal ring segments; PIOL, phakic intraocular lens; SE, spherical equivalent; CDVA, corrected-distance-visual-acuity; UDVA, uncorrected-distance-visual-acuity.

To date, several protocols of CXL plus have been developed and implemented:

- TG-PRK (max 80 μm) then high-fluence CXL (known as the Athens protocol) (218)
- tPTK then CXL (known as the Cretan protocol) (219)
- tPTK then PRK + CXL (known as the Cretan Plus protocol) (220)
- Selective transepithelial TG-PRK with simultaneous accelerated CXL (STARE-X) (221)
- CXL + ICRS (203)
- CXL + ICRS + transepithelial TG-PRK (214)
- CXL + ICRS + toric phakic IOL + TG-PRK (222)
- CXL + ICRS + TG-tPRK (I-TRESK/CXL) (217).

With the recent advances in refractive surgery and the tools available to refractive surgeons, it is now more common for patients who are having CXL plus procedures to have customized treatment targeting wavefront guided HOAs with no compromise to optical zones. This ablation pattern allows regularization of the cornea, overcoming their otherwise inefficient optical system to allow for an improvement in their functional vision.

The timing of ICRS implantation, in the context of combined CXL, is still being debated, though in clinical setting it is not always a choice to make. Often patients may have already undergone CXL, other times they have had ICRS but are progressing. There is a logic that ICRS should precede CXL as it may be easier to insert the rings from a biomechanical point of view (202). Same day simultaneous ICRS and CXL has also gained support (223). In a recent randomized clinical trial evaluating the timing of CXL and ICRS for keratoconus (*n* = 198 eyes of 198 patients), Hersh et al. (203) reported a similar safety and effectiveness between concurrent surgery (same day for ICRS then CXL) and sequential surgery (ICRS then CXL 3 months later). Overall, there was a mean improvement in UDVA (by two logMAR lines), CDVA (by 1.1 logMAR line), and maximum topographic keratometry (by 7.5 D).

**FIGURE 6:**
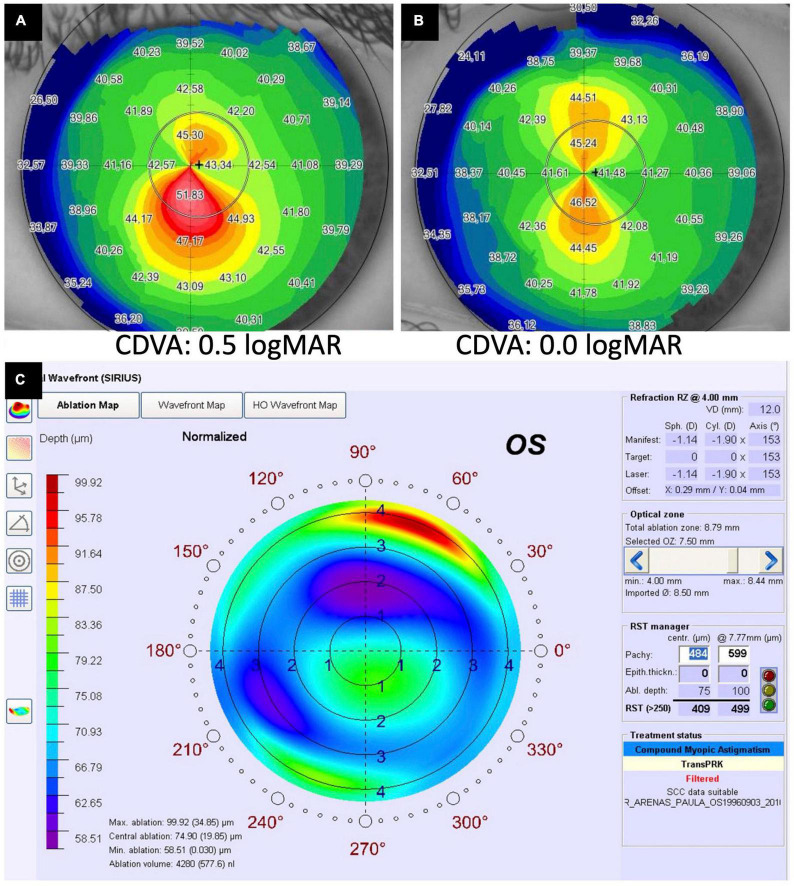
A case of corneal cross-linking (CXL) plus [with wavefront transepithelial photorefractive keratectomy (tPRK) followed by simultaneous same-day conventional CXL] for treating moderate progressive keratoconus. **(A)** Preoperative corneal topography demonstrating a significant irregular astigmatism [with high inferior-superior (IS) asymmetry] and a corrected-distance-visual-acuity (CDVA) of 0.5 logMAR. **(B)** Postoperative corneal topography demonstrating a significant improvement in irregular astigmatism and CDVA to 0.0 logMAR following the treatment. **(C)** The ablation map demonstrating the setting of the tPRK used in this patient.

Several studies have demonstrated that combined PRK and CXL are able to achieve significant improvement in vision and corneal regularity when compared to CXL alone, with long-term stabilization of the disease progression (Figure 6) (205, 210). However, combined PRK with CXL may result in a deeper penetration of the UV-A radiation during CXL [potentially attributed to the ablation of the Bowman’s layer (BL)], which needs to be considered during the preoperative planning to avoid any undesirable damage to the corneal endothelium (210). In addition, the timing of PRK in relation to CXL, whether to be performed simultaneously or sequentially (where PRK is performed 6–12 months post-CXL), is another important clinical question. Several factors need to be considered during combined PRK + CXL. First, the ablation effect of PRK on crosslinked corneas may differ from non-crosslinked corneas, potentially affecting the predictability and outcome of the combined treatment if performed sequentially (i.e., CXL then PRK). Second, the crosslinked corneal tissues (particularly the anterior portion of the stroma) are removed by the PRK if performed later, which may increase the risk of progression of keratoconus in the long-term. Third, the risk of postoperative stromal haze may be different between two approaches. CXL is known to induce apoptosis of the stromal keratocyte (224), which may reduce the risk of postoperative haze formation if PRK is applied simultaneously. With a sequential approach, the crosslinked stroma becomes repopulated by host keratocytes, which may result in a higher rate of postoperative haze formation.

Kanellopoulos et al. (208) previously conducted a clinical study comparing the outcome of same-day simultaneous versus sequential CXL and TG-PRK in 325 eyes with progressive keratoconus. Although both approaches offered beneficial effects, the same-day simultaneous approach was shown to exhibit significantly better improvement in UDVA, CDVA, refraction, and topographic keratometric measurements, with lesser risk of postoperative stromal haze. Recently, selective transepithelial topography-guided photorefractive keratectomy combined with accelerated corneal crosslinking (STARE-X) protocol has been described to achieve good corneal regularity and improve both visual acuity and corneal aberrations at 2 years after the procedure (221).

Corneal stromal haze remains a recognized postoperative risk in combined CXL and PRK, especially in cases with sequential approach (208) and in patients with a history of atopy (225). Intraoperative topical mitomycin C (MMC) 0.02% is commonly used to reduce the risk of corneal haze following PRK (226) and is also used in the Athens protocol (i.e., TG-PRK followed by high-fluence CXL) (209). However, a study showed that topical MMC may increase the risk of stromal haze following CXL, for which the mechanism is poorly understood (227). On the other hand, the Cretan protocol does not use any MMC intraoperatively and the rate of stromal haze is similar to those that undergo CXL alone.

Multiple CXL plus procedures such as CXL, ICRS and PRK showed benefit regardless of the sequence of events. However, the common approach is to first perform ICRS implantation followed by combined PRK/PTK and CXL (Table 4) (214, 216, 228). Surgical success is dependent on patient selection, intraoperative factors such as the proper depth of ring pavement, and reliability of the nomogram used. As CXL leads to gradual changes in the anterior corneal curvature for up to a year postoperatively, there is a valid question over the exact refraction target in combined procedures. Nonetheless, promising results are still observed in specific protocols without any adjustment for such refractive changes (229).

Early detection and treatment of patients with aggressive medical management of co-existing atopic disease, avoidance of eye rubbing, and CXL in an early stage of the disease may reduce the need for additional refractive procedures (230, 231). On the other hand, management of cases of moderate to advanced disease is more challenging, and as refractive surgery continues to advance, it is envisaged that customized CXL-keratorefractive surgery tailored to each individual’s need and disease severity is likely to take place in the future (232–234).

### 4.4. Stromal keratophakia (allogeneic)

Tissue addition techniques or keratophakia has been described as a vision correction technique for aphakia, hyperopia, and myopia via the change of the anterior corneal curvature (235–237). To allow for the addition of a donor lenticule into the stroma, the host stromal pocket needs to be fashioned, either with microkeratome or with FSL. However, the use of microkeratome has been shown to produce inconsistent anatomic and refractive outcomes (238), which explains the lack of uptake of this approach. Jonas et al. (239) first explored and demonstrated the feasibility of FSL in safely preparing the host bed for intrastromal lamellar keratoplasty without disturbing the recipient’s corneal epithelium and BL, using an *ex vivo* porcine corneal model. Subsequently, Tan et al. (240) described a new technique named “intralamellar keratoplasty,” which is a type of stromal additive keratoplasty that involves the insertion of a laser-fashioned donor stromal lenticule into an FSL-assisted intrastromal pocket created in the host cornea. They reported an improvement in refractive and topographic astigmatism (by 1–2 D) and CL tolerance (50% with successful CL fitting) at 6 months follow-up.

More recently, Mastropasqua et al. (241) implanted +8 D hyperopic lenticules from donor corneas into corneas with stage III and IV keratoconus with central cones. The negative meniscus lenticules were inserted at a depth of 120 mm and centered on the apex of the cone. The study showed significant cornea flattening, asphericity reduction, and corneal biomechanical strength improvement. It has been shown that the lenticule addition into the stroma leads to stromal remodeling, corneal flattening and restoration of epithelial thickness (242). Similarly, Alio Del Barrio et al. (243) reported an innovative stromal keratophakia technique using 9 mm diameter, 120 μm thick, FSL-assisted lenticules of decellularized human donor corneal stroma, with or without autologous adipose-derived adult stem cells (ADASCs) recellularization, for treating advanced keratoconus. At 6 months postoperative, there was an improvement in UDVA, CDVA, refractive spheres, HOAs, and corneal thickness. Decellularization of the donor corneal stroma helps reduce the risk of allograft corneal stromal rejection. In addition, corneal stromal cell density is shown to increase following the implantation of the decellularized lenticules, especially when they are recellularized with ADASCs, at 1-year postoperative (244).

Several other stromal keratophakia techniques have also been described. In a case reported by Pradhan et al. (245), a 400 μm stromal lenticule was prepared by performing a DALK on a donor cornea. Excimer laser ablation was then done to remove 50 μm of the tissue to help regularize the host cornea. This lenticule was inserted in a patient with progressive keratoconus and thinnest pachymetry of 425 μm. The intrastromal space was created by combining LASIK and DALK, leaving 125 μm of anterior stromal tissue and 140 μm of posterior tissue. At 1 year follow-up, the authors reported a reduction of steepest keratometry (by >7 D) and Q value (by 0.6). Successful tissue additive procedures have also been reported in pediatric patients with advanced keratoconus (246, 247). Jadidi and Mosavi (248) successfully implanted planar lenticules of thickness 140–250 μm at a depth of 250 μm in a series of four patients with advanced keratoconus. The technique of stromal keratophakia with excimer laser-assisted donor keratomileusis has also been described (249), with improvement in the mean simulated keratometric values and myopia.

Stromal addition to the mid-peripheral cornea represents another approach for stromal keratophakia. Ganesh and Brar (250) described the use of cryopreserved stromal lenticules derived from patients undergoing SMILE to create a donut-shaped lenticule. This lenticule was soaked in riboflavin and inserted in ectatic cornea at a depth of 100 μm centered on the first Purkinje image, followed by simultaneous accelerated CXL. This procedure, termed as femtosecond intrastromal lenticule implantation (FILI), results in tissue addition in the mid-periphery. At 6 months follow-up, the authors reported a significant improvement in UDVA, CDVA, Q values, and corneal aberrations in 5 eyes with moderate keratoconus. One eye with advanced keratoconus did not show improvement which led the authors to conclude that FILI worked best for patients with keratometry values <58 D.

In addition, a recent meta-analysis of 10 studies by Riau et al. (251) demonstrated that FSL-assisted stromal keratophakia serves as a feasible technique to expand corneal volume, correct refractive errors, and regularize corneal curvature in patients with keratoconus. However, further studies are required to help standardize the technique, including the choice of lenticules (concave vs. convex), the need and timing for combined CXL, and mathematical modeling to account for the long-term epithelial changes and stromal modeling that will have an important impact on the outcome of the technique.

### 4.5. Keratoplasty

As discussed above, keratoconus can be managed conservatively with spectacles and/or CL and corneal interventions such as CXL, ICRS, and keratophakia. With disease progression, visual correction with these measures may become insufficient or infeasible. It is estimated that up to 20% of patients with keratoconus will eventually need a keratoplasty (252–254).

Keratoconus is the leading indication for keratoplasty worldwide but the trend varies among different countries. In European countries such as France and Ireland, nationwide studies reported that keratoconus is their top indication for keratoplasty, accounting for around 19% cases (255, 256). In New Zealand, 28% of all keratoplasty were performed for eyes with keratoconus in 2019 (257). In a study specifically looking at the trend of PK in US, keratoconus accounted for 16% of their transplantation cases (11). However, studies conducted in Asia (e.g., China and Singapore) demonstrated a lower proportion of keratoplasty for keratoconus (∼10% of the national corneal transplantation activity) (258, 259), highlighting the geographical variations in the indication for keratoplasty and potentially the prevalence of keratoconus.

For more than 100 years, PK was the only surgical option for patients with keratoconus. However, over the past two decades, there has been a paradigm shift in the surgical technique from PK to DALK for treating advanced keratoconus worldwide (10, 11, 255–257, 259–262). Moreover, a new technique, Bowman’s layer transplantation (BLT), has recently been proposed to treat advanced keratoconus and delay or reduce the need for DALK or PK (263, 264).

In view of the potential complications and the shortage of donor corneas, keratoplasty is usually reserved as the last resort for treating advanced keratoconus. In general, DALK is the technique of choice when Descemet’s membrane remains free of scarring or opacity and corneal endothelium remains healthy (10, 43, 253, 254, 265–267). On the other hand, PK is indicated when there is significant, full-thickness corneal scarring (e.g., following acute corneal hydrops or perforation), extremely thin cornea (<150–200 μm, though technically is still possible for DALK), or in cases with co-existing endothelial dysfunctions (43, 265, 266).

#### 4.5.1. Penetrating keratoplasty

Despite being out favored by newer techniques, PK is well-established and long-term data has shown that PK has a high success rate in managing advanced keratoconus. The Australian Corneal Graft Registry Study (one of the most extensive corneal transplant studies which included 4,834 eyes) reported a relatively favorable long-term outcome for PK performed for keratoconus, with a graft survival rate of 89 and 49% at 10 and 20 years postoperative, respectively (268). This is similarly to the finding observed in the recent Singapore Corneal Transplant Study where the PK survival rate was 44% at 20 years (258). In addition, 74% of the eyes that had undergone PK for advanced keratoconus achieved a CDVA of 20/40 or better at their last follow-up as compared to only 8% of the eyes preoperatively (268). Several other studies also showed that long-term visual acuity after PK can improve to 20/30–20/40 with only spectacle correction (269–271). However, as many of the keratoconus patients undergo PK at a relatively young age, it is likely that these patients will require more than one graft in their lifetime, for which the risk of graft rejection is increased with repeated grafts (272, 273).

Graft rejection is the main reason for graft failure in PK, and it occurs most frequently in the first few years after PK, with around 50% of graft rejection occurring within the first year and 90% within the first 4 years (268). Recurrence of corneal ectasia, usually at the graft-host junction, is another recognized cause for “graft failure” in long-term surviving PK where the graft is anatomically clear but not functionally useful (268). The risk is estimated at 10–30% at 10–20 years postoperative and is higher in more advanced keratoconus (274–276). Significant post-keratoplasty astigmatism represents another important factor that may limit the visual outcome despite an anatomically clear graft. In the Australian Corneal Graft Registry Study, 61% of the patients who had PK needed refractive correction with spectacles and/or CL, and significant astigmatism (≥5 D) was prevalent among 77% of the patients (268). In addition, acute hydrops may also manifest following post-PK in keratoconic patients (277).

#### 4.5.2. Anterior lamellar keratoplasty

In advanced keratoconus without any previous acute corneal hydrops, DALK with big bubble techniques is the most common type of lamellar keratoplasty performed, accounting for more than 50% of the corneal transplantations in some countries (43, 266, 278). There are many other ALK techniques, such as manual layer-by-layer pre-descemetic DALK (pdDALK), dDALK with viscodissection, pdDALK with the Melles technique, and FSL-assisted DALK. Still, all of these are less commonly performed (43, 254, 265, 267, 278, 279). With the demonstration of the Dua’s layer/pre-Descemet’s layer (PDL) and the associated Type-1, Type-2, and mixed big bubbles, the different planes of cleavage achieved during DALK are now well-defined (280, 281).

DALK has become the treatment of choice because it has several advantages over PK. With DALK, the host corneal endothelial cells are retained, eliminating the risk of corneal endothelial rejection and graft failure (253, 254, 265, 266, 282). Other benefits of DALK include lower risk of endophthalmitis, reduced dependence on long-term topical corticosteroids [hence lesser risk of ocular hypertension (OHT) and glaucoma], faster recovery, and stronger graft-host junction (282, 283).

Literature comparing the clinical outcomes of PK and DALK has shown inconclusive results. In a French Study, it was found that the predicted graft survival period of DALK was almost three times of PK (49.0 vs. 17.3 years) (284). This difference is possibly explained by the higher endothelial cell density and elimination of the risk of endothelial rejection after DALK (267, 283, 285). However, a Cochrane review, which only included two randomized controlled trials (RCTs), concluded that there was no difference in CDVA, graft survival or keratometric outcomes between PK and DALK for keratoconus (286), though the findings were of low evidence due to small sample size. These results were supported by two subsequent systematic reviews and meta-analyses, which demonstrated no significant difference in graft survival and keratometric astigmatism between PK and DALK (283, 285). However, in terms of BCVA at 6 months or longer post-transplantation, these two studies showed conflicting results, with one showing DALK being superior to PK and vice versa (283, 285). This difference can be possibly explained by the heterogeneity of DALK techniques employed in the included studies. For instance, DALK with a successful big bubble technique may lead to a superior visual acuity and quality of vision compared to manual dissection technique, as the latter technique could result in more graft-host interface irregularity and undesirable ocular aberrations, especially when the residual stromal thickness is high (267, 287, 288). However, similar postoperative results have been demonstrated between big-bubble and manual dissection techniques when the latter achieves relatively thin residual stromal host bed (289). More importantly, studies have consistently demonstrated a lower graft rejection in DALK than PK (283, 285).

Long-term use of topical corticosteroids has been linked to OHT and/or glaucoma. It was reported that up to 46% of post-PK keratoconic eyes experienced OHT (290–293). With a lower requirement of topical steroid in DALK, eyes following DALK are less prone to developing OHT or glaucoma. Studies have shown that only 1–36% of keratoconic eyes develop OHT after DALK, with a significantly lower risk of developing secondary glaucoma than post-PK (278, 293–296). Compared to PK, DALK may offer a faster rehabilitation postoperatively due to stronger graft-host junction (297), which allows for earlier suture removal (without affecting the graft-host junction) and reduces the risk of suture-related post-keratoplasty infectious keratitis (298, 299). In addition, the risk of recurrence of keratoconus in the graft is considerably less common after DALK than PK (275, 300).

However, DALK is associated with a relatively steep learning curve and the surgical outcome is highly surgeon-dependent. A retrospective London study found that intraoperative perforation of DM occurred in 45.4% of eyes, and their conversion rate to PK was 24.1% (278). This considerably higher complication rate was likely attributed to a relative lack of relevant surgical experience among surgeons. Most cases were performed by trainee surgeons, and none of the surgeons in this study conducted more than 20 DALK cases per year (278). In other previous studies where more than 100 cases were performed by high-volume corneal surgeons, a considerably lower complication rate was reported, with ∼10% intraoperative micro/macroperforation of the Descemet membrane and ∼0.3–3% conversion rate to PK (265, 286, 288, 301, 302). Intra-operative imaging and use of assistive techniques such as FSL may improve the consistency and outcomes of DALK in keratoconus (303–306).

In addition to the graft-host junction (which is along the circumference as with PK), DALK has another graft-host interface which exists between the anterior surface of the host DM or PDL and the posterior surface of the transplanted donor tissue. This interface corresponds to the diameter/surface area of the donor tissue. This graft-host interface provides a plane for implantation of debris intraoperatively, interface haze, the spread of ingrowing blood vessels, inflammatory debris and organisms following suture-related infections. These issues are not seen with PK but do not override the advantages of DALK.

#### 4.5.3. Bowman’s layer transplantation

Bowman’s layer fragmentation is seen early in the course of keratoconus and it is a sensitive and specific indicator of disease (307–309). It is postulated that further deterioration of vision or disease progression can be prevented by replacing the BL. Isolated BLT for 10 eyes with advanced keratoconus (ineligible for conservative treatments such as CXL or ICRS) was first attempted by Van Dijk et al. (263). The same group further expanded the cohort to include 22 eyes in 2015, with no intra- or post-operative complications reported in these two studies (263, 310). At 6-month post-operatively, the mean maximum keratometry (K max) reduced by 6–8 D and remained stable after that with a mean follow-up period of 21 months (263, 310). The mean best spectacle-corrected visual acuity (BSCVA) improved from 1.27 to 0.90 logMAR post-operatively, with a stable contact lens-corrected-visual-acuity (CLVA) (310). No cases of progression of keratoconus, graft rejection, or allograft reaction were reported (310). These promising early results sparked interest among surgeons in performing BLT as an alternative to PK or DALK for eyes with advanced keratoconus.

Similar results were reported in a later study of BLT of 15 eyes with advanced keratoconus, with a mean BSCVA improving from 1.35 logMAR preoperative to 0.96 logMAR at 12 months postoperative (311). It was also found that the corneal HOAs, especially spherical aberration, improved on both anterior and posterior corneal surfaces after BLT (311). There are also studies which modify the original techniques such as with the use of FSL or intraoperative optical coherence topography (iOCT), to improve the reproducibility of BLT by aiming to reduce the risk of stromal perforation (312, 313). However, Tong et al. (313) found that with the help of iOCT, their results were no better than those achieved without iOCT.

Long-term follow-up of the original cohort of eyes that underwent BLT reported that the estimated success rate of BLT at 5 years was 84% (264). Kmax continued to be stable at 5 years after the initial drop postoperatively (264, 314). At 5–7 years follow-up, BSCVA also remained stable after the initial improvement but CLVA did not improve as compared to preoperatively (264, 314). No complications were reported except three acute corneal hydrops developed in between 4.5 and 6.5 years postoperatively, and these two patients had a history of atopy and severe eye rubbing (264, 314, 315).

This limited evidence to date suggests BLT may preserve the CDVA in patients with advanced keratoconus, potentially serving as an alternative to PK or DALK. One limitation of the adoption of BLT as treatment for advanced keratoconus is the technical difficulty but successful attempts from surgeons around the world have supported the feasibility of this technique (264, 311, 314, 316). More studies are needed to further determine the long-term efficacy and safety of BLT in treating advanced keratoconus.

## 5. Future directions

### 5.1. Corneal stromal regeneration

Keratoconus is a corneal disease that primarily affects the corneal stroma and BL, with these structures being severely diseased in advanced cases (309). Thus, current research is partially focusing on whether advanced keratoconic eyes can be rehabilitated by minimally invasive procedures that efficiently regenerate the corneal stroma to improve vision and reduce the risk of complications associated with PK or DALK (317–320).

**FIGURE 7:**
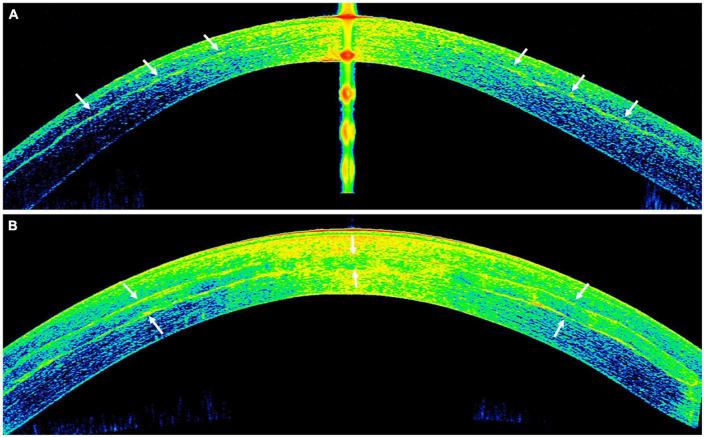
Anterior segment optical coherence tomography (AS-OCT) images demonstrating the outcome of implanted autologous adipose derived adult stem cells (ADASCs) in advanced keratoconus. **(A)** An ASOCT image showing the effect of the cellular therapy of the corneal stroma by an intrastromal implantation of autologous ADASCs in a patient with advanced keratoconus at 6 months post-treatment. Observe the hyperreflective band of neo-collagen (around 15 μm thickness) at the level of the stromal pocket (arrows). **(B)** An ASOCT image showing the corneal stromal enhancement by an intrastromal implantation of a decellularized lamina of human corneal stroma (arrows) colonized with autologous ADASC in a patient with advanced keratoconus at 12 months post-treatment.

Recently, several studies have reported the preliminary safety and efficacy results of corneal stromal stem cell therapy for patients with advanced keratoconus (243, 321, 322). Autologous mesenchymal stem cells (MSCs), in the form of suspension containing quiescent autologous adipose derived adult stem cells (ADASCs; obtained by elective liposuction), were transplanted into a mid-stroma FSL-assisted lamellar pocket in patients with advanced (stage IV) keratoconus (321). They could confirm, in a clinical setting, what was previously demonstrated in the animal model (323). ADASCs were capable of surviving *in vivo*, showing a perfect biointegration without any clinical inflammatory response or rejection, generating new collagen production within the corneal stroma (Figure 7A), and improving the vision. However, the creation of the stromal pocket may induce some aberrometric and keratometric changes, partially interfering with visual improvement (321). Moreover, they reported a case where preoperative stromal scars at the cone apex improved after the implantation of such MSC. This correlates well with the acquired knowledge from the experimental data in animals, which demonstrates the potential of stem cell therapy in alleviating pre-existing mild stromal scars (324, 325). Nevertheless, according to the clinical and pre-clinical available evidence, the direct intrastromal implantation of MSCs within the cornea achieves the production of new extracellular matrix but is not expected to be quantitatively enough to restore the thickness of a severely thin human cornea (like in severe keratoconus). On the other hand, this approach may provide a promising treatment modality for corneal dystrophies, and for the modulation of corneal scars (320).

In this context, the addition of decellularized human corneal sections repopulated by autologous MSC with the purpose of enhancing the anatomic rehabilitation of the keratoconic cornea has also been studied (243, 326). Both experimental and clinical studies have demonstrated good biointegration of such implants within the host corneal stroma, mimicking the normal natural strength and transparency of cornea with no risk of rejection reported so far (Figure 7B). *In vivo* confocal biomicroscopy studies have also shown that the transplanted MSCs can survive and differentiate into corneal keratocytes, while the decellularized implants show cellular repopulation starting from 3 months postoperative (244). Long-term studies (up to 3 years) demonstrated a moderate efficacy (a mean visual improvement by two Snellen lines) and a moderate flattening effect, with no reported postoperative complications (325). This innovative technique may also help reduce the need for donor human corneas as one donor corneal tissue can be used for up to three patients (324).

Nevertheless, the presented data is so far preliminary and limited to small samples, though additional studies are underway. Moreover, MSCs have proven to exhibit immunomodulatory properties on even xenogeneic transplants, though this capacity differs among different individuals (324). This raises the question about the ideal approach, since the autologous MSCs will carry the same genetic defects that precipitated the disease on first instance, and so their use may increase the risk of disease recurrence in long-term. The creation of cellular banks that could select the best donor cells with the greater biological effect may enhance these initial clinical outcomes. The future will bring further answers that can help establish this stromal cellular therapy as a potential therapeutic alternative to keratoplasty in some early cases of keratoconus.

### 5.2. Stromal keratophakia (xenogeneic)

In the previous section “4.4. Stromal keratophakia (allogeneic),” we highlighted the use of allogeneic stromal keratophakia as a novel treatment for keratoconus to obviate the need for DALK and PK. However, this technique is dependent on the availability of human donor corneas, which is currently limited by the global shortage. To overcome this issue, Rafat et al. (327) recently described an innovative xenogeneic stromal keratophakia technique using a “bioengineered cell-free porcine construct, double cross-linked (BPCDX).” The porcine construct is made of medical-grade type 1 porcine dermal collagen (to mimic the human corneal stroma with abundant type 1 collagen) and is double cross-linked to improve the strength and stability of the implantable hydrogel. In a recently completed phase 1 open-label clinical trial of 20 patients with advanced keratoconus, the authors reported a mean improvement in the corneal thickness (by 200–300 μm) and visual acuity (final vision was around 20/30–20/60) and a decrease in maximum keratometry (by ∼11–14 D), with no adverse event, over a 24-month follow-up period. These promising results highlight the potential of xenogeneic stromal keratophakia, using BPCDX, as a safe, minimally invasive and donor-independent technique for advanced keratoconus. This may also serve as a useful alternative to the current conventional keratoplasty and overcome the barrier of the shortage of human donor corneas.

### 5.3. Gene therapy

The recent advent of next generation sequencing (NGS) has significantly advanced the ability to accurately sequence any genome of interest with reduced time and cost (328). This technology has enabled the detection of many previously unknown genetic mutations and has greatly deepened the understanding of various diseases (329). In addition, identification of these genetic mutations may serve as important biomarkers to predict the severity and progression of the disease and open the door to gene therapy targeting the underlying mutations to prevent or deter the disease progression (329).

Emerging studies have demonstrated important genetic implications on the pathogenesis and progression of keratoconus (330, 331). Lu et al. (332) previously conducted a meta-analysis of >20,000 individuals in European and Asian populations and identified two central corneal thickness-associated loci, FOXO1 and FNDC3B, that are strongly linked to the development of keratoconus. Several genome-wide linkage studies (GWLS) and genome-wide association studies (GWAS) have mapped out a wide array of genetic mutations linked to keratoconus. These include *LOX* gene (which encodes for lysyl oxidase, an enzyme that is involved in the cross-linking of extracellular proteins), *COL5A1* gene (which encodes for collagen type V). *TGFBI* gene (which encodes for transforming growth factor beta induced protein), *ZNF469* gene (which regulates corneal collagen structure and synthesis), *VSX1* gene (which encodes for visual system homeobox 1), and many others (333). So far, gene therapy has demonstrated promise as a novel therapy for treating a wide range of inherited retinal degeneration (334, 335). It is possible that the successful development and translation of these retinal gene therapies will pave the way for similar gene therapy for many other ocular diseases (with genetic predisposition) in the future, including keratoconus. However, as keratoconus is a polygenic disease, the development of effective gene therapy for keratoconus will undoubtedly be more complex than for monogenic disease like certain inherited retinal degeneration (336).

### 5.4. Artificial intelligence

Over the past decade, there has been an explosion of artificial intelligence (AI) research in healthcare, primarily owing to the significant improvement in computer processing power, advancement in deep learning techniques, and increased availability of big data, electronic health records and open-source databases (337–342). The development of AI-power platforms and telemedicine in healthcare, including ophthalmology, was further fueled by the recent COVID-19 pandemic to address the unprecedented rise in the healthcare backlog and to reduce the need for conventional face-to-face consultation as part of the containment and mitigation strategy (343–346).

To date, AI has demonstrated its potential for the diagnosis of keratoconus (347–351). Furthermore, clinical applicability in the management of keratoconus, ranging from early detection of the disease, including sub-clinical keratoconus (or forme fruste keratoconus), preoperative screening and prediction of postoperative ectasia following keratorefractive surgery, and guiding and predicting the need for surgery (352–356). As corneal tomography (e.g., Oculus Pentacam) and AS-OCT (e.g., swept-source CASIA) are two most common modalities used in screening and diagnosing keratoconus (357), these images are most commonly utilized to train and develop the AI algorithms for keratoconus. So far, various AI approaches have been described and used, including artificial neural network (ANN), random forest, automated decision-tree classification, support vector machine (SVM) learning, convolutional neural network (CNN), and unsupervised learning (351, 352, 358–368).

Yousefi et al. (369) previously developed an unsupervised AI algorithm using swept-source AS-OCT images (CASIA) of ∼3,000 eyes to identify the stages and severity of keratoconus. The algorithm successfully identified four clusters of patients (ranging from normal eyes, forme fruste keratoconus to advanced keratoconus), and correlated well with the Ectasia Status Index. KeratoDetect, a CNN-based algorithm, has been shown to be able to automatically distinguish keratoconic eyes from normal eyes using topographic maps obtained from Scheimpflug imaging (370). By using 3,000 corneal topographic images (with a mixture of healthy cornea and keratoconus images), the algorithm was able to accurately diagnose keratoconus with 99.3% accuracy. Similarly, Chen et al. (351) developed a CNN-based algorithm to accurately detect and grade keratoconus based on the color-coded Scheimpflug topography maps. More recently, Gao et al. (360) developed an artificial neural network, named KeratoScreen, based on Zernike coefficient obtained from Scheimpflug corneal tomography. By using images of 208 patients, the algorithm was able to accurately distinguish normal eyes from subclinical keratoconus and keratoconus in >95% cases. AI has also been shown to be able to efficiently predict local and global progression of keratoconus based on Pentacam parameters, which may facilitate an earlier treatment for keratoconic eyes that are at higher risk of progression (371).

In addition, AI has demonstrated its usefulness in guiding implantation of ICRS in keratoconic corneas and predicting surgical outcomes. Valdés-Mas et al. (368) proposed an ANN based on multilayer perceptron to predict the postoperative improvement in corneal curvature and astigmatism following ICRS, with a predictive error of less than 1 D for both parameters. Another study demonstrated that an ANN-based algorithm was able to guide the ICRS implantation better than the manufacturer’s nomogram and resulted in better visual outcome and less HOAs (372). It is also likely that AI may be used to optimize the planning for CXL-plus treatment (e.g., CXL + PRK) and improve the visual and refractive outcomes (233). Furthermore, based on ∼12,000 ASOCT images, Yousefi et al. (356) were able to develop an accurate unsupervised AI system that may help identify patients with corneal disease who are at a higher risk for future keratoplasty, including DALK for keratoconus.

## 6. Conclusion

The management of keratoconus has evolved significantly over the past century. As with most diseases, the approach has evolved from treating the disease to preventing and early diagnosis. The advent of CXL has rendered it possible to halt the progression of keratoconus. Recent years have seen several modifications of CXL to extend the treatment eligibility to thinner corneas. Efforts have been made to combine CXL with keratorefractive procedures to regularize the cornea and improve visual and refractive outcomes. While not yet widely performed, allogeneic stromal keratophakia and BLT have shown favorable short- to mid-term results. DALK has emerged as the preferred choice of keratoplasty over PK (when DM/PDL is not affected), and autologous stem cells and BPCDX are being investigated to avoid keratoplasty and its associated complications and reduce/eliminate the need for human donor corneas.

## 7. Methods of literature search

Electronic databases, including MEDLINE and EMBASE, were searched to identify relevant studies on the management of keratoconus. Only English articles were included in this review article. Key words used were “keratoconus,” “corneal ectasia,” “contact lens,” “corneal cross-linking,” “intracorneal ring segment,” “keratorefractive surgery,” “keratoplasty,” “stromal keratophakia,” “corneal stromal regeneration,” “gene therapy,” “artificial intelligence,” and “machine learning.” The bibliographies of included articles were manually screened to identify further relevant studies. The final search was last updated on 31 December 2022.

## Author contributions

DT: study conceptualization and supervision. DT, RD, ZO, RR, and JA: literature review, data collection and curation, and drafting of initial manuscript. AB, MA, JM, DS, HD, and RA: critical revision of manuscript. All authors: data analysis, interpretation, and final approval of manuscript.
